# Small Molecule Inhibitors of DYRK1A Identified by Computational and Experimental Approaches

**DOI:** 10.3390/ijms21186826

**Published:** 2020-09-17

**Authors:** Hye Ree Yoon, Anand Balupuri, Kwang-Eun Choi, Nam Sook Kang

**Affiliations:** Graduate School of New Drug Discovery and Development, Chungnam National University, 99 Daehak-ro, Yuseong-gu, Daejeon 34134, Korea; hyeree7775@naver.com (H.R.Y.); balupuri@cnu.ac.kr (A.B.); hwendiv@naver.com (K.-E.C.)

**Keywords:** DYRK1A, molecular docking, molecular dynamics simulation, topological water network

## Abstract

Dual-specificity tyrosine phosphorylation-regulated kinase 1A (DYRK1A) is a protein kinase with diverse functions in cell regulation. Abnormal expression and activity of DYRK1A contribute to numerous human malignancies, Down syndrome, and Alzheimer’s disease. Notably, DYRK1A has been proposed as a potential therapeutic target for the treatment of diabetes because of its key role in pancreatic β-cell proliferation. Consequently, DYRK1A is an attractive drug target for a variety of diseases. Here, we report the identification of several DYRK1A inhibitors using our in-house topological water network-based approach. All inhibitors were further verified by in vitro assay.

## 1. Introduction

The phosphorylation of proteins catalyzed by protein kinases plays a major role in the regulation of cellular processes, such as cell proliferation, differentiation, apoptosis, and signal transduction. Abnormal protein phosphorylation has been implicated in several diseases. Consequently, protein kinases have emerged as major drug targets [1]. Dual-specificity tyrosine phosphorylation-regulated kinase 1A (DYRK1A) belongs to the DYRK family of kinases, which includes four other members (DYRK1B, DYRK2, DYRK3, and DYRK4). This kinase regulates critical cellular processes, including the proliferation and differentiation of neuronal progenitor cells [2]. DYRK1A is abnormally expressed in Down syndrome, Alzheimer′s disease, Pick′s disease [3], lung cancer, cervical cancer, gastrointestinal stromal tumors (GIST), glioblastoma, melanoma, acute megakaryoblastic leukemia, acute lymphoblastic leukemia, and acute myeloid leukemia [4,5,6,7]. Recently, DYRK1A was found to be involved in human pancreatic β-cell proliferation, making it a potential therapeutic target for the treatment of Type 1 and Type 2 diabetes [8,9,10,11,12]. Insufficient pancreatic β-cell mass or function leads to diabetes mellitus. Under high glucose conditions, β-cells increase intracellular calcium (Ca^2+^) levels. This activates calcineurin, which in turn dephosphorylates the nuclear factor of activated T-cells cytoplasmic (NFATc) proteins. The dephosphorylation and activation of NFATc lead to nuclear import. Nuclear NFATc kinases, such as DYRK1A and GSK3β phosphorylate NFATc proteins, cause them to undergo nuclear export. The inhibition of DYRK1A and GSK3β blocks NFATc nuclear export and increases β-cell proliferation [9,13].

DYRK1A has attracted great attention as a potential therapeutic target because of its role in neurodegenerative diseases, various cancers, and diabetes. Over the last several years, considerable research has been conducted to identify and develop novel DYRK1A inhibitors. A number of DYRK1A inhibitors from different sources have been reported in the literature. Natural products, such as harmine and its analogues (β-carbolines) [14], leucettines [15], benzocoumarins [16], quinalizarine [17], epigallocatechin and other flavan-3-ols [18], the peltogynoids Acanilol A and B [19], and indolocarbazoles (staurosporine, rebeccamycin and their analogues) [20] are known to inhibit DYRK1A. Small molecule DYRK1A inhibitors identified by drug discovery efforts include INDY [21], GNF4877 [9], DANDY [22], FINDY [23], amino-quinazolines [24], pyrazolidine-diones [25], meriolins [26], pyridine and pyrazines [27], chromenoindoles [28], 11*H*-indolo (3,2-c)quinoline-6-carboxylic acids [29], CC-401 [12], 5-iodotubercidin [8], thiazolo [5,4-f]quinazolines (EHT 5372) [30], indole-3-carbonitriles [31], and thiadiazines [32]. However, none are currently in clinical trial. Here, we report the identification of novel DYRK1A inhibitors using an integrated computational and experimental approach. Our research group has developed an algorithm to determine topological water networks (TWNs) [33,34]. Previously, we used the TWN-based approach to investigate kinase selectivity [33], protein–ligand binding [34], and drug repositioning between kinases [35] to design kinase inhibitors [35,36], explore protein folding [37], and understand protein hydration [38]. In our previous report, 26 kinase-staurosporine complex structures were used for TWN analysis and staurosporine-based repositioning [35]. We identified a kinase with staurosporine-sensitive activity similar to that of DYRK1A. Through kinase TWN analysis, GSK3β, with low-binding site similarity but a high distribution of water molecules at the C site, was identified. In the present study, we used TWN analysis and known GSK3β inhibitors to identify new DYRK1A inhibitors.

## 2. Results

**Figure 1:**
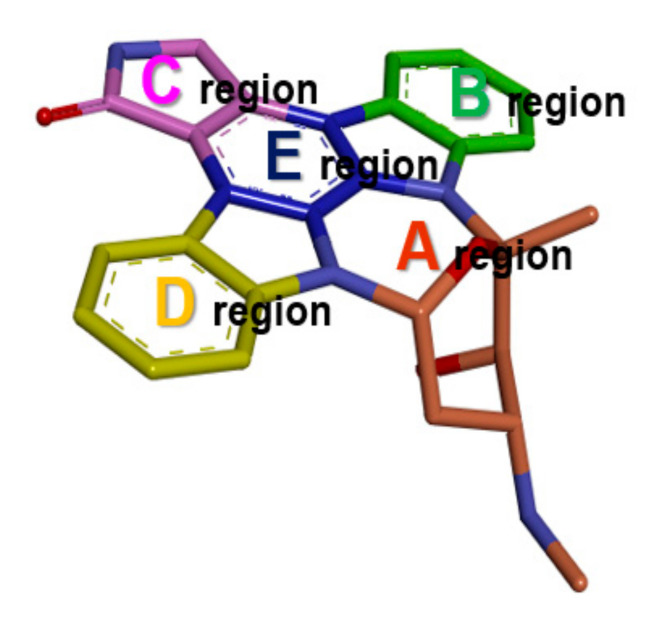
Structure of staurosporine. ATP binding site of DYRK1A and GSK3β was divided into five regions (A–E) based on staurosporine’s binding mode. Various regions occupied by the ligand are highlighted in different colors (A: wheat, B: green, C: pink, D: yellow, E: blue).

We performed MD simulations on the DYRK1A and GSK3β structures in the apo state, and analyzed TWNs in their binding site. Staurosporine is a potent pan-kinase inhibitor [39]. The planar structure of staurosporine with few rotatable bonds allows it to occupy the adenosine triphosphate (ATP) binding sites of kinases. The co-crystal structure of human GSK3β, in complex with staurosporine (PDB code 1Q3D), is available [40]. However, the co-crystal structure of DYRK1A, in complex with staurosporine, had not yet been determined. We therefore obtained the crystal structure of human DYRK1A (PDB code 4YLL) [29] and docked staurosporine into its ATP binding site. For TWN analysis, we divided the ATP binding site into five regions (A–E), based on staurosporine’s binding mode (Figure 1).

### 2.1. DYRK1A vs. GSK3β

**Figure 2:**
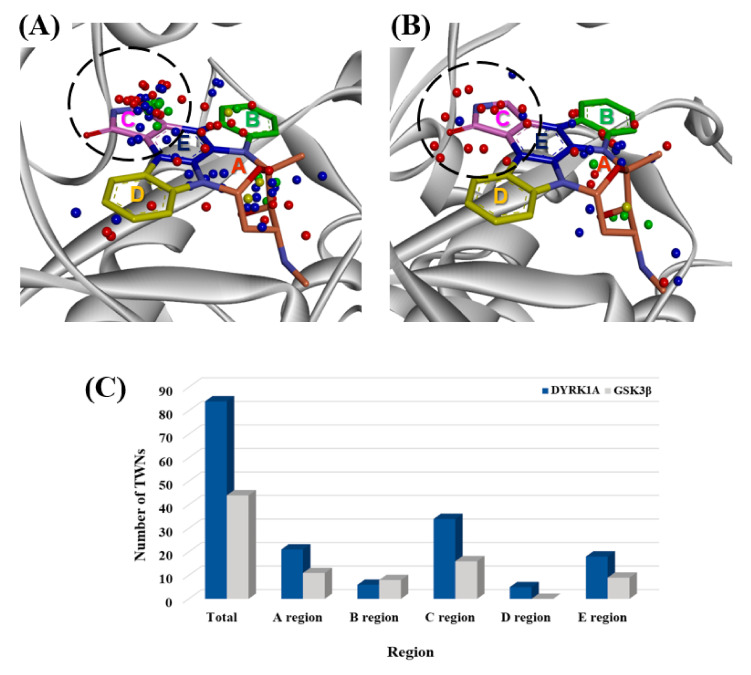
Superposition of staurosporine (pink stick model) with the center of mass of TWNs for (**A**) DYRK1A and (**B**) GSK3β. Center of masses of R3, R4, R5, and R6 TWNs are shown as red, blue, green, and yellow spheres, respectively. TWNs within the C region of the ATP binding site are highlighted with a sky-blue circle. (**C**) Distribution of TWNs are in various regions of the ATP binding site.

DYRK1A is a dual-specificity kinase that possesses both serine/threonine and tyrosine kinase activities, while GSK3β is a serine-threonine kinase. Both DYRK1A and GSK3β have been implicated in diabetes [9,13]. As shown in Table 1, these kinases do not have a high total sequence similarity or binding site similarity. Total sequence similarity and binding site similarity were found to be 44.1% and 32.0%, respectively. Staurosporine exhibits comparable activity against these kinases. It showed IC_50_ values of 19 and 15 nM against DYRK1A [20] and GSK3β [41], respectively. Sequence and binding site similarities were not able to account for the comparable activities of staurosporine against DYRK1A and GSK3β. Thus, staurosporine was extracted from the co-crystal structure of GSK3β (PDB code 1Q3D) and re-docked into the ATP binding site. DYRK1A and GSK3β docking results were compared. Although they exhibited similar IC_50_ values, the binding energies differed by more than −10 kcal∙mol^−1^. Staurosporine displayed a binding energy of −68.9 and −79.0 kcal∙mol^−1^ for DYRK1A and GSK3β, respectively. We therefore analyzed TWNs in the ATP binding sites of these kinases. This analysis revealed a high percentage of TWNs in the C regions (hinge region) of both kinases (Table 1 and Figure 2). DYRK1A exhibited 40.5% TWNs while GSK3β demonstrated 36.4% TWNs in the C region. The hinge regions of kinases are known to play a key role in ligand binding. Furthermore, we calculated the TWN–ligand shape similarity and observed comparable values of 54% and 61% for DYRK1A and GSK3β, respectively. Based on the TWN results, we anticipated that known GSK3β inhibitors with a high percentage of TWNs in the C region and reasonable TWN−ligand shape similarities could be repositioned as DYRK1A inhibitors.

### 2.2. TWN-Based Repositioning

**Figure 3:**
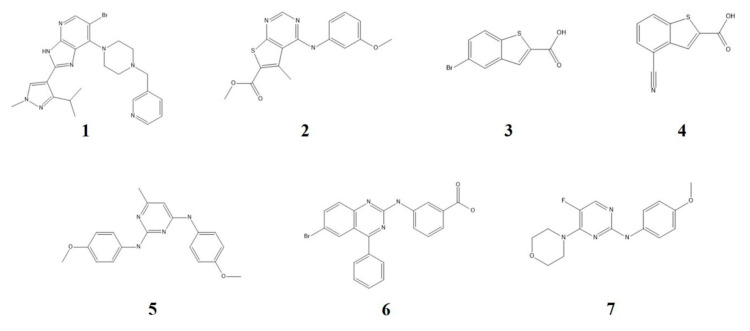
Structures of the selected compounds obtained from the in-house library screening.

Compounds AZD1080 [42] and SB-415286 [43] are known GSK3β inhibitors with IC_50_ values of 31 and 78 nM, respectively. These compounds were docked into the binding site of DYRK1A, and TWNs were analyzed around the ligands. AZD1080 and SB-415286 showed binding energies of −58.3 and −83.9 kcal∙mol^−1^, respectively (Table 2). Similar to staurosporine, both compounds displayed a high percentage of TWNs in the C region (hinge region) of DYRK1A. AZD1080 showed 61.8% TWNs, while SB-415286 showed 47.2% TWNs in the C region. Docking results revealed that both compounds formed hydrogen bonds with hinge residues Glu239 and Leu241, which occupy the C region. Additionally, they showed hydrogen bond interactions with Lys188 (Figure 3). These residues are known to play important roles in DYRK1A kinase activity and in the binding of DYRK1A inhibitors [14,44]. We calculated TWN–ligand shape similarity for the compounds and obtained reasonable similarities of 36% and 44% for AZD1080 and SB-415286, respectively. Compounds AZD1080 and SB-415286 are available commercially. We purchased compounds AZD1080 and SB-415286 and verified their DYRK1A inhibitory activities by in vitro assay. In accordance with the TWN–ligand shape similarity values, SB-415286, with an IC_50_ of 445 nM, was found to be more potent than AZD1080 (IC_50_ = 2911 nM).

### 2.3. Docking and TWN-Based Screening

Our results suggest that compounds with a high percentage of TWNs in the C region and reasonable TWN–ligand shape similarity could inhibit DYRK1A. We docked our in-house compounds into the binding site of DYRK1A to identify potential ligands. Structures of the selected ligands (compounds **1**−**7**) are displayed in Figure 3. Binding energy and TWN results are provided in Table 2. The selected compounds showed a higher percentage of TWNs in the C region compared with the other regions. Except for compound **6**, all compounds showed TWNs of more than 50% in the C region. We calculated TWN–ligand shape similarity for these ligands. Compounds **5** and **7** showed low similarity (<25%). Similar to AZD1080 (36%), compounds **1**, **2** and **6** exhibited moderate similarity (25−40%). Similar to staurosporine (54%) and SB-415286 (44%), compounds **3** and **4** showed high similarity (>40%). We verified DYRK1A inhibitory activities of these compounds by in vitro assay. In accordance with the TWN–ligand shape similarity, compounds **5** and **7** displayed low DYRK1A inhibition (<40%) at 10 μM. Similar to AZD1080 (71%), compounds **1**, **2** and **6** exhibited moderate inhibition (40–80%). However, in contrast to SB-415286 (91%), compounds **3** and **4** did not show high DYRK1A inhibition. Compound **3** demonstrated 82% inhibition, whereas compound **4** exhibited 71% inhibition. Structural analysis revealed that compounds **3** and **4** are smaller compared with the other compounds. They could not cover the A and D regions because of their small size. This might be the reason for their moderate inhibition values, despite the high TWN–ligand shape similarity. Based on the percentage inhibition data, IC_50_ values against DYRK1A were calculated for all the selected compounds, except compounds **5** and **7**. The activity values are provided in Table 2.

## 3. Discussion

DYRK1A phosphorylates NFAT, thereby inhibiting the effects of calcium signaling and maintaining NFAT in an inactive state [45]. DYRK1A is a negative regulatory factor of the cell cycle that promotes the G0 state or conversion to differentiation. In malignant cells, DYRK1A promotes cell survival by inhibiting pro-apoptotic signaling [46].

Recently, DYRK1A was found to be associated with human pancreatic β-cell proliferation [10,32]. Type 1 and type 2 diabetes are characterized by a decrease in pancreatic β-cell mass [9]. β-cells in high glucose conditions increase intracellular calcium levels. Activation of calcineurin leads to dephosphorylation of NFATs and subsequent nuclear translocation. As nuclear NFATc kinases, DYRK1A and GSK3β phosphorylate NFATc proteins to induce nuclear export. Both DYRK1A and GSK3β are negative regulators of the NFAT pathway, and this pathway is fundamentally important for β-cell proliferation [9,13].

Our research group has developed and is optimizing algorithms for determining TWNs. In our previous study, we performed MD simulations on 26 kinases in aqueous solution and analyzed TWNs in their ATP binding sites [35]. Other previous studies used TWN analysis to study protein hydration [38], kinase selectivity [33], kinase inhibitor design [35,36], and drug repositioning [35].

The purpose of this study was to identify DYRK1A inhibitors by screening our in-house library using TWN–ligand shape similarity based on known inhibitors of other kinases with TWN patterns similar to DYRK1A. Through multiple kinase TWN analyses, GSK3β with low binding site similarity, but high distribution of water molecules at the C region compared to DYRK1A, was selected. Notably, GSK3β participates in the same signaling pathway as DYRK1A, and their staurosporine inhibition values are similar. The GSK3β inhibitors AZD 1080 and SB-415286 were tested for their IC_50_ values against DYRK1A. The binding energy and TWN–ligand shape similarity were also analyzed. The IC_50_ values revealed better inhibition by SB-415286 than AZD1080 against DYRK1A. Compounds were selected from the in-house library based on the IC_50_ values and TWN–ligand shape similarity of staurosporine, AZD 1080, and SB-415286 against DYRK1A. In the TWN analysis, compounds that account for more than 35% of the C region were selected through screening. The IC_50_ values for compounds with TWN–ligand shape similarity values of less than 25% were not determined. Compounds with TWN–ligand shape similarities of 25–40% showed IC_50_ values of 2.9–6.8 μM. Compounds with more than 40% TWN–ligand shape similarity showed IC_50_ values of less than 0.5 μM. However, compounds **3** and **4** deviated from these trends, with TWN–ligand shape similarity values over 50%, but IC_50_ values of about 3.2 μM. We investigated the underlying cause of the deviation by analyzing the binding mode predicted through molecular docking studies. The analysis revealed that because the compound sizes were small, they could not extend in the direction of the hinge (Glu239, Leu241) or the key residue (Lys188), which is key to kinase competitive inhibition. These compounds nonetheless have inhibitory activity and are suitable for use as a scaffold. To develop more effective inhibitors from these compounds, optimization is required by adding functional groups in the direction of the hinge region as well as key residues. In a future study, we plan to optimize the TWN–ligand shape similarity range and compare the electro-shape similarities.

In this paper, we described how to identify a DYRK1A inhibitor from another known inhibitor using the TWN–ligand shape similarity method. As a computational drug discovery method, we propose the TWN–ligand shape similarity method through TWN analysis as a way to rapidly identify compounds amenable to drug repositioning. The TWN–ligand shape similarity method can be used to search for target compounds by acquiring scaffolds through high throughput screening (HTS) and prediction of biological activity.

## 4. Materials and Methods

### 4.1. Protein Preparation

The X-ray crystal structures of human DYRK1A (PDB code 4YLL) [29] and human GSK3β (PDB code 1Q3D) [40] were downloaded from the Protein Data Bank (PDB) and all non-protein molecules were discarded. These structures were further processed using the Prepare Protein module in Discovery Studio 2017 (BIOVIA, San Diego, CA, USA). This process included the identification of missing residues, addition of hydrogen atoms, assignment of bond orders, and formal charges. Protonation states were assigned under the assumption that the systems were at a pH of 7.4.

### 4.2. Molecular Dynamics (MD) Simulation

GROMACS is a freely available, versatile package to perform MD simulation. It is extremely fast and supports all the usual algorithms you would expect from a modern MD implementation. It provides extremely high performance compared to other programs and contains quite a few features that make it stand out from the competition. It is user-friendly, with a fully automated topology builder for proteins. There is plenty of consistency checking, and clear error messages are issued if something is incorrect. It can be run in parallel and can write MD trajectory data in a very compact way. There is no need to write any code to perform usual MD analysis, as it provides many flexible tools for the analysis [47]. Due to these reasons, we selected GROMACS for the MD simulation. MD simulations were performed with GROMACS 4.5.3 [47]. Protein topology was generated using CHARMM27 force field [48]. Protein was solvated in a cubic box using the TIP3P water model [49]. Counter ions were added to ensure the neutrality of the system. Then, the system was subjected to energy minimization using 500 steps of the steepest descent algorithm. This was followed by equilibration over two stages. Firstly, the system was equilibrated in the NVT ensemble (constant number of particles, volume, and temperature) for 0.1 ns, followed by equilibration in the NPT ensemble (constant number of particles, pressure, and temperature) for 0.2 ns. NVT equilibration was executed at a temperature of 300 K using V-rescale thermostat [50], whereas NPT equilibration was carried out at a pressure of 1 bar using the Parrinello–Rahman barostat [51]. Finally, a production run was performed for 10 ns with a time step of 1 fs. Periodic boundary conditions (PBC) were applied to the system. In MD simulation, PBC are usually applied to avoid problems with boundary effects. PBC make it possible to simulate a small system that is not terminated by a surface, as it is periodically repeated in all directions [47]. A linear constraint solver (LINCS) algorithm was used to constrain bonds [52]. The LINCS algorithm provides a high performance and it is faster and more stable than other constraint algorithms [47]. A cut-off distance of 1.2 nm was used for all short-range non-bonded interactions, while long-range electrostatics were calculated using the Particle mesh Ewald method [53]. After MD simulation, 100 trajectory files were extracted for TWN analysis.

### 4.3. Topological Water Network (TWN) Analysis

Water molecules form water-ring networks through hydrogen bonds, which the authors have termed topological water networks (TWNs) [33,34,35,36,37,38]. These networks include small rings, such as trimers (R3), tetramers (R4), pentamers (R5), and hexamers (R6). The potential functions considered in the TWNs involve a rigid TIP3P water model. The interactions between water molecules are conveniently modeled using Lennard–Jones and Coulomb potentials [49]. The interactive potential energy between two water molecules (*a* and *b*) is expressed by the Equation (1) below:

(1) $$v\left(a,b\right)=\overset{on\;a}{\underset{i}{\sum }}\overset{on\;b}{\underset{j}{\sum }}\frac{q_{i}q_{j}e^{2}}{r_{ij}}+\frac{A}{r_{oo}{}^{12}}-\frac{C}{r_{oo}{}^{6}}$$

where,

v(*a*, *b*) = interaction potential energy
*r_oo_* = distance between oxygen atoms
*q_i_* = partial charge on the *i* site (−0.834*e*)
*q_j_* = partial charge on the *j* site (0.417*e*)
*r_ij_* = distance between *q_i_* and *q_j_*
*A* = repulsive force between *i* and *j* (582,000 kcal∙Å^12^ mol^−1^)
*C* = attractive force between *i* and *j* (595 kcal∙Å^6^mol^−1^)

Parameters were chosen in such a way that they produced reasonable structural and energetic results for liquid water. The energy criterion of −2.25 kcal∙mol^−1^ was used to determine hydrogen bonding between water molecules. This value was selected as a criterion because it closely corresponds to the minimum value of the water–water pair potential energy distribution [49].

### 4.4. Binding Site Similarity

Binding site similarity was calculated using the geometric hashing method [54]. This method compares a set of binding sites quickly. The algorithm identifies equivalent heavy atoms between binding sites and matches them in the same relative spatial orientation. Binding site similarity is expressed by the following Equation (2):

(2) $$R_{3}=\frac{n_{match}}{n_{site1}+n_{site2}-n_{match}}$$

where *R*_3_ represents the similarity score. It takes into account the total size of the two binding sites (*n_site_*_1_ and *n_site_*_2_). It is calculated analogously to the Tanimoto coefficient, and its value ranges from 0 to 1. A value of 1 indicates the self-comparison of a binding site. The *n_match_* denotes the number of atoms comprising the largest possible matching [55].

### 4.5. TWN-Ligand Shape Similarity

Shape similarity was calculated using the ultrafast shape recognition (USR) method [56]. This method is based on the assumption that the relative position of atoms defines the shape of a molecule. The molecular shape is described by a set of one-dimensional distributions with three-dimensional shape information. The USR method uses the distributions of all the atomic distances to four different reference locations: the molecular centroid (*ctd*), the farthest atom to *ctd* (*fct*), the closest atom to *ctd* (*cst),* and the farthest atom to *fct* (*ftf*). The first three moments from each of the four one-dimensional distributions are considered to describe a molecule, as in Equation (3):

(3) $$\vec{M}=\left(\mu 1^{ctd},\mu 2^{ctd},\mu 3^{ctd},\mu 1^{cst},\mu 2^{cst},\mu 3^{cst},\mu 1^{fct},\mu 2^{fct},\mu 3^{fct},\mu 1^{ftf},\mu 2^{ftf},\mu 3^{ftf}\right)$$

Shape similarity is estimated by the Equation (4) below:

(4) $$S_{qi}={\left(1+\frac{1}{12}\overset{12}{\underset{l=1}{\sum }}|M\begin{array}{c} q\\ l \end{array}-M\begin{array}{c} i\\ l \end{array}|\right)}^{-1}$$

where *S_qi_* is the similarity score function, ${\vec{M}}^{q}$ and ${\vec{M}}^{i}$ are the vectors of shape descriptors for the query and the *i*th screened molecule, respectively.

### 4.6. Molecular Docking

Crystal structures of proteins were obtained and processed as described in the protein preparation section. Molecular docking studies were performed on the processed structures using the LigandFit module [57] of Discovery Studio 2017 (BIOVIA). The Prepare Ligand protocol was used to build and optimize ligands. Partial charges were assigned using the Momany–Rone partial charge method. Energy minimization was carried out with the CHARMM force field. The binding site was defined based on the co-crystallized ligand. For each ligand, 50 docked poses were generated and scored using scoring functions. Protein–ligand interactions were considered for selecting the binding modes of the ligands.

### 4.7. Procurement, Synthesis and Characterization

Compound AZD1080 (2-hydroxy-3-(5-(morpholinomethyl)pyridin-2-yl)-1*H*-indole-5-carbonitrile) and compound SB-415286 (3-((3-chloro-4-hydroxyphenyl)amino)-4-(2-nitrophenyl)-1*H*-pyrrole-2,5-dione) were purchased from Selleckchem (Houston, TX, USA). Compound **1** (6-bromo-2-(3-isopropyl-1-methyl-1*H*-pyrazol-4-yl)-7-(4-(pyridin-3-ylmethyl)piperazin-1-yl)-3*H*-imidazo(4,5-b)pyridine) was synthesized and characterized as reported in our previous work [58]. Compound **2** (methyl 4-((3-methoxyphenyl)amino)-5-methylthieno (2,3-*d*)pyrimidine-6-carboxylate) was purchased from Otava Ltd. (Vaughan, Canada). Compound **3** (5-bromobenzo[*b*]thiophene-2-carboxylic acid) and Compound **4** (4-cyanobenzo[*b*]thiophene-2-carboxylic acid) were purchased from Ambinter (Orléans, France). Compound **5** (*N*^2^,*N*^4^-bis(4-methoxyphenyl)-6-methylpyrimidine-2,4-diamine), compound **6** (3-((6-bromo-4-phenylquinazolin-2-yl)amino)benzoic acid) and compound **7** (5-fluoro-N-(4-methoxyphenyl)-4-morpholinopyrimidin-2-amine) were purchased from VitasMLab (Causeway Bay, Hong Kong).

### 4.8. In Vitro Assay

Enzymatic assays were performed by Eurofins Scientific Inc. Korea (Brussels, Belgium). DYRK1A(h) was incubated with 8 mM MOPS pH 7.0, 0.2 mM EDTA, 50 μM RRRFRPASPLRGPPK, 10 mM MgAcetate, and (γ–33P–ATP (specific activity approx. 500 cpm/pmol, concentration as required). The reaction was initiated by the addition of the MgATP mix. After incubation for 40 min at room temperature, the reaction was stopped by the addition of 3% phosphoric acid solution. Then, 10 μL of the reaction was then spotted onto a P30 filtermat and washed three times for 5 min in 75 mM phosphoric acid and once in methanol prior to drying and scintillation counting. IC_50_ was calculated for inhibitors, including staurosporine (from 10mM DMSO stock solution), depending on various final concentrations. All assays were performed in duplicate, and the average IC_50_ value was reported.

## 5. Conclusions

In conclusion, we identified inhibitors of DYRK1A using a computational TWN-based approach, and we subsequently verified their inhibitory activity experimentally. More potent DYRK1A inhibitors can be developed through further optimization of these molecules.

## Figures and Tables

**Table 1:** Comparison of DYRK1A and GSK3β.

	DYRK1A	GSK3β
PDB code	4YLL	1Q3D
Sequence similarity (%)	−	44.1
Binding site similarity (%)	−	32.0
Staurosporine IC_50_ (nM)	19	15
Binding energy (kcal∙mol^−1^)	−68.9	−79.0
TWN−ligand shape similarity (%)	54.0	61.0

**Table 2:** Summary of TWNs, Binding Energy and In Vitro Results for the Screened Compounds **1–7** against DYRK1A.

	1		2		3		4		5		6		7	
	Distribution of TWNs													
Region	Number	Ratio (%)	Number	Ratio (%)	Number	Ratio (%)	Number	Ratio (%)	Number	Ratio (%)	Number	Ratio (%)	Number	Ratio (%)
A	11	19.3	9	14.3	0	0.0	0	0.0	0	0.0	17	28.8	0	0.0
B	0	0.0	6	9.5	6	14.6	6	14.6	0	0.0	5	8.5	6	13.0
C	31	54.4	34	54.0	26	63.4	21	51.2	15	57.7	21	35.6	28	60.9
D	4	7.0	0	0.0	0	0.0	0	0.0	1	3.9	4	6.8	1	2.2
E	11	19.3	14	22.2	9	22.0	14	34.2	10	38.5	12	20.3	11	23.9
Total	57	100.0	63	100.0	41	100.0	41	100.0	26	100.0	59	100.0	46	100.0
TWN–ligand shape similarity (%)	30.0		33.0		52.0		52.0		19.0		29.0		22.0	
Binding energy (kcal∙mol^−1^)	−41.9		−79.6		−83.8		−87.3		−52.3		−82.3		−56.3	
% inhibition at 10 μM	70		58		82		71		27		76		33	
IC_50_ values (nM)	6767		5712		3246		3240		N.D.		5833		N.D.	

N.D.: not determined.
